# Evaluation of Blood-Brain-Barrier Permeability, Neurotoxicity, and Potential Cognitive Impairment by *Pseudomonas aeruginosa*'s Virulence Factor Pyocyanin

**DOI:** 10.1155/2022/3060579

**Published:** 2022-03-17

**Authors:** Muhammad Ibrahim Rashid, Habiba Rashid, Saadia Andleeb, Amjad Ali

**Affiliations:** ^1^Department of Industrial Biotechnology, Atta Ur Rahman School of Applied Biosciences, National University of Sciences and Technology, Islamabad, Pakistan; ^2^Department of Molecular Biology & Genetics, Institute of Basic Medical Sciences, Khyber Medical University, Peshawar, Pakistan; ^3^Department of Biotechnology, Virtual University of Pakistan, Peshawar, Pakistan; ^4^Department of Anatomy, Institute of Basic Medical Sciences, Khyber Medical University, Peshawar, Pakistan

## Abstract

Pyocyanin (PCN) is a redox-active secondary metabolite produced by *Pseudomonas aeruginosa* as its primary virulence factor. Several studies have reported the cytotoxic potential of PCN and its role during infection establishment and progression. Considering its ability to diffuse through biological membranes, it is hypothesized that PCN can gain entry into the brain and induce oxidative stress across the blood-brain barrier (BBB), ultimately contributing towards reactive oxygen species (ROS) mediated neurodegeneration. Potential roles of PCN in the central nervous system (CNS) have never been evaluated, hence the study aimed to evaluate PCN's probable penetration into CNS through blood-brain barrier (BBB) using both in silico and in vivo (Balb/c mice) approaches and the impact of ROS generation via commonly used tests: Morris water maze test, novel object recognition, elevated plus maze test, and tail suspension test. Furthermore, evidence for ROS generation in the brain was assessed using glutathione S-transferase assay. PCN demonstrated BBB permeability albeit in minute quantities. A significant hike was observed in ROS generation (*P* < 0.0001) along with changes in behavior indicating PCN permeability across BBB and potentially affecting cognitive functions. This is the first study exploring the potential role of PCN in influencing the cognitive functions of test animals.

## 1. Introduction

**Figure 1:**
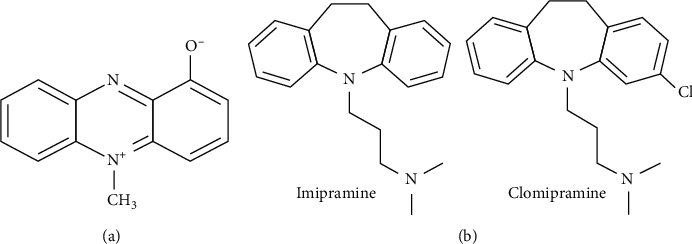
(a) Pyocyanin molecular structure. (b) Tricyclic antidepressants (TCAs) share great deal of structural similarity with PCN. TCAs are blood-brain barrier permeable and regulate neurotransmitters in the synapse.

*Pseudomonas aeruginosa* is a ubiquitous nosocomial infectious agent. It causes the clinical significance of this bacterium intensified due to the phenomenon of its natural tendency for acquiring drug resistance mechanisms [1–5]. Infections from superbug strains of *P. aeruginosa* in immune-compromised and transplant patients are becoming a very serious healthcare issue ([6], Abd [7, 8]). *P. aeruginosa* produces many primary and secondary metabolites with multiple bioactivities. Prominent of them is pyocyanin (PCN) (Figure 1(a)) [6, 9–15]. It has two benzene rings and a heterocycle in the middle ([16–18], Cole, and Taylor, 1986).

Being a redox-active compound PCN alters the redox equilibrium inside a biological system. It has been detected in higher quantities in sputum samples of *P. aeruginosa* infected cystic fibrotic patients, up to 100 *μ*M [19]. These higher levels indicate the vital role PCN plays at the site of infection. Inside the lungs, studies have reported that PCN not only interacts with tissues of the lung but also with the cells of the immune system [20, 21]. Cytotoxicity of PCN is attributed to its ability to generate ROS in particular H_2_O_2_ which induces oxidative stress in biological systems. Upon detection of PCN inside a cell, cells utilize their NAD (P) H to avoid the damage caused by reactive oxygen species (ROS)/oxidative stress. But NAD (P) H non-enzymatically reduces PCN which, in presence of molecular oxygen, reacts with it to produce superoxide and hydrogen peroxide after dismutation. As a result, cells lose their stockpiles of energy to be generated in the electron transport chain and become starved while the damage due to oxidative stress and ROS continues to mount ([22] and Newman, 2007). Ranging from cardiac and neurological disorders to chronic lung and liver diseases, oxidative stress is considered one of the important disease pathologies [21, 23–28].

A study by Venugopal et al. reported the PCN mediated induction of oxidative stress in hippocampal cells and subsequent reversal of the redox activity due to N-acetyl-L-cysteine [29, 30]. Arora et al. have explored the systemic level inflammation and impact of PCN mediated toxicity on murine behavioral activity [31]. The study found PCN to be affecting the locomotor activity of the subjects as well as their immobility behavior. PCN has also been used to induced ROS in human neural progenitor cells (hNPCs), mediated its oxidative stress levels using gintonin-enriched fraction [32].

Interestingly, tricyclic antidepressants (TCA), a class of CNS medication shares key structural features with PCN. TCA (i.e., clomipramine, imipramine, and desipramine) are able to cross BBB [33–37]. These serve as neurotransmitter regulators in the synapse [38–40]. This structural similarity between PCN and TCA provided the ground work for this study (Figure 1(b)).

**Figure 2:**
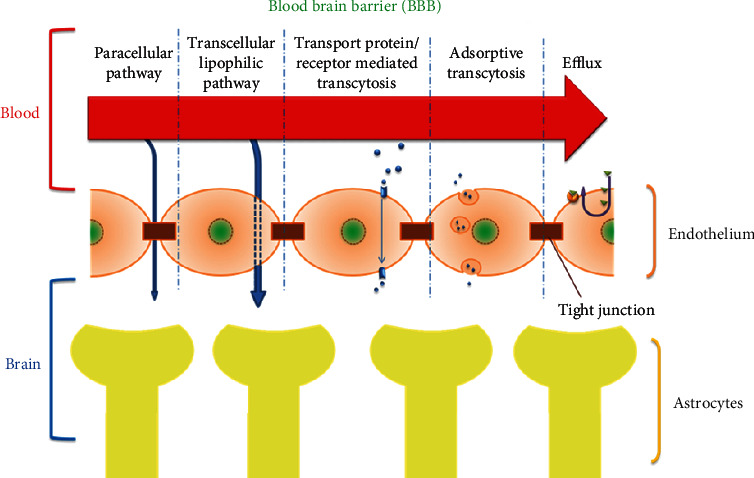
Schematic depiction of entry routes into and across the blood-brain barrier.

Despite the abundance of *P. aeruginosa* in clinical settings, potential roles of PCN have never been evaluated in the central nervous system (CNS) in animal models. The possible routes of entry into the brain across BBB are depicted in Figure 2. Once inside the brain, it might induce oxidative stress and potentially play a role in the development of neurodegenerative disorders, as it has long been established that most neurodegenerative disorders involve the induction of oxidative stress in the CNS [25, 41].

This study is focused on the unveiling of any potential roles played by PCN in the development of neurodegenerative disorders and management of the damages caused by PCN during *Pseudomonas aeruginosa* infection. Aims of the study are the following: (1) prediction of BBB permeability of PCN via in silico approaches using PCN zwitterionic structure at blood pH and confirmation by HPLC, (2) assessment of redox activity across the BBB, and (3) evaluation of neurotoxicity and potential impact on cognitive functions. For achieving these objectives, a comprehensive set of computational approaches was adapted to study PCN movement into the brain. BBB permeability was initially studied computationally. Simulated approaches were adopted to better understand how PCN crosses the BBB. These approaches employed physio-chemical and structural properties of PCN. This structural information was retrieved from ChEMBL and PubChem databases. Conclusions of dry lab experiments were validated in the balb/c murine model. Being a redox-active compound, the effects of oxidative stress were evaluated to foresee any possible role in neurodegeneration by measurement of oxidative stress responses. Brain oxidative stress levels were evaluated twice as much in comparison to control non-PCN injected mice. The elevated levels of oxidative stress in the brain yielded the validation of the dry lab results. Potential neurotoxicity was studied by conducting behavioral tests.

## 2. Materials and Methods

### 2.1. Online Servers and Databases Accessed for In Silico Analyses

Chemical and structural information of PCN were obtained from Pubchem (CID:6817) and ChEMBL (CHEMBL2289232) databases [42, 43]. Servers utilized for blood-brain barrier permeability assessment: the blood-brain barrier (BBB) predictor server (http://www.cbligand.org/BBB/index.php). This server employs AdaBoost (LiCABEDS) and SVM algorithms combining with 4 different fingerprints (MACCSFP(M.)(M.)(M.)(M.)(M.)(M.)(M.)(M.)(M.)(M.)(M.)(M.)(M.)(M.)(M.)(M.)(M.)(M.)(M.)(M.)(M.)(M.)(M.)(M.), OpenbabelFP2(O)(O)(O)(O)(O)(O)(O)(O)(O)(O)(O)(O)(O)(O)(O)(O)(O)(O)(O)(O)(O)(O)(O)(O), Molprint2DFP [44], and PubChemFP [45]) to predict BBB permeability of a given compound. PreADMET server (http://preadmet.bmdrc.kr/adme/) uses two different classification systems for assessment of CNS activity/inactivity degree of absorption into CNS [46]. PreADMET server also provided different in silico toxicity evaluation tools (i.e., human intestinal absorption, in vitro MDCK cell permeability, in vitro skin permeability, plasma protein binding, and in vitro Caco2 cell permeability). Way2Drug server (http://www.way2drug.com/geb/) was used to calculate LogBB value for PCN [47]. SwissADME server (http://www.swissadme.ch/) was also used for BBB permeability assessment [48]. PCN is reported to be in a zwitterionic state at the blood's alkaline pH [28]. Such PCN structure was built using Canonical SMILES (CN1C2 = CC = CC = C2N = C3C1 = CC = CC3 = O) obtained from PubChem and was submitted to each server unless it was built online depending upon servers' requirement.

### 2.2. Animals, Groups, and PCN Dosage

Naïve adult balb/c mice (aged 3–4 months), obtained from the National Institute of Health Islamabad, were used in the study. Mice were maintained at ambient light (14 hours) and dark (10 hours) cycle, with ad libitum food and water. Male and female mice were placed in separate cages with 4 animals in each cage. Mice behavioral tests were performed in the light cycle (09:00–16:00 hours). Pyocyanin (PCN: Sigma-P0046) was purchased from Sigma Aldrich and stored at 4°C. PCN was dissolved in deionized water for injection administration, fresh each day. Animals were used for three sets of experiments. For HPLC analysis mice were divided into two groups; (1) control group (*n* = 4) which received intraperitoneal (IP) injection of normal saline and (2) test group (*n* = 4) which was administered PCN IP injection to achieve 50 *μ*mol/mL systemic concentration. For glutathione S-transferase analysis, mice were randomly divided into two groups; (1) control group (*n* = 4) receiving normal saline IP injection and (2) test group (*n* = 4) receiving PCN IP injection to achieve 50 *μ*mol/mL systemic concentration. For behavioral tests, animals were divided into three groups: (1) PCN control group (*n* = 8) received a single IP injection of normal saline, (2) PCN I group (*n* = 8) received a single IP injection to achieve 100 *μ*M/mL PCN systemic concentration, and (3) PCN II group received IP injections of PCN to maintain 50 *μ*M/mL systemic concentration for five days. Total blood volume was calculated for each mouse, using the following formula [49]:

(1) $$\begin{array}{c} \text{Blood volume}\left(\text{mL}\right)=0.06\times \text{body weight}\left(\text{g}\right)+0.77. \end{array}$$

For groups PCN control and PCN I, a single injection was administered to each animal before commencing the tests, while for PCN-II injections were administered for 5 days before the test to study the effects of prolonging PCN exposure. Injections were administered 30 min before the behavioral tests. Animal model studies were approved by the Institutional Review Board (Ref. # ASAB/IRB-83).

### 2.3. In Vivo Assessment of PCN BBB Permeability

Balb/c mice (*n* = 8) were IP injected with PCN (50 *μ*mol/mL systemic conc.) 12 hrs before brain harvesting. Perfusions were performed for each subject animal. Whole brains were harvested and homogenized (1 g in 2 mL PBS buffer) using sonication, sonicator settings were timer 30 seconds and pulse cycle for 5 sec amplitude <40%. The resultant homogenized suspension was subjected to centrifugation at 15000 × *g* for 10 min, the pellet was discarded and the supernatant was subjected to PCN extraction via chloroform-acidified water extraction [50]. Extracts were subjected to high-performance liquid chromatography analysis using C18 reserves phase column (250 mm). Mobile phase *Acetonitrile*: *TFA (100: 0.04)* for 5 min and *Acetonitrile: TFA: H_2_O (60: 0.04: 40)* for 25 min. Detection was made using UV visible detector at 280 nm wavelength [51].

Levels of oxidative stress in tissues were calculated to the activity of glutathione S-transferase enzyme's (GST) overtime in tissues to total proteins of organ homogenate. The protein concentration was determined by observing optical density at 280 nm (OD_280_). For the estimation of protein 100 *μ*l of supernatant was added into a test tube containing 900 *μ*l phosphate buffer (100 mM, pH 7.0). Using these protein concentration readings, a standard curve was developed. For detection of oxidative stress in the brain via GST assay, the same injection dosage and sample homogenization procedure were adopted except mice were euthanized 24 hrs postinjection administration. GST activity was assayed using 1-chloro-2,4-dinitrobenzene (CDNB) as substrate, based on the method of Habig et al. 1974 [52]. The specific activity expressed as nMol of CDNB-GSH conjugate produced per minute per mg of protein.

#### 2.3.1. Behavior Tests

### 2.4. Morris Water Maze Test

**Figure 3:**
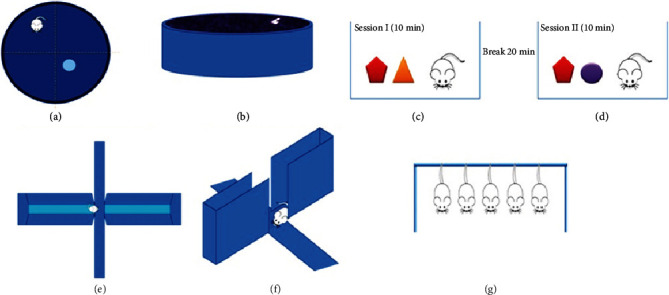
Schematic representations of behavioral tests performed. Morris water maze (a and b), novel object recognition (c and d), elevated plus maze (e and f), and tail suspension (g).

Mice were trained to find a submerged platform (13 cm diameter and 34 cm high). The platform was placed in a steel pool (120 cm diameter × 60 cm height). The pool was filled with opaque water up to 35 cm so that the platform is 1 cm merged below the water surface as described [53]. Mice underwent 25 training trials in 5 days for finding the hidden platform. Time taken by the mice to reach the platform was noted and analyzed (Figures 3(a) and 3(b)).

### 2.5. Novel Object Recognition

Novel object recognition test was conducted in a square chamber (40 × 40 × 40 cm) as described [53]. For training, mice were introduced to the chamber and left for habituation after 5 min two objects (*X* + *Y*) were placed within the arena. The interaction session was allowed for 10 min after which the mouse was removed from the chamber, and the objects were washed with 70% ethanol after each mouse. After a break of 20 min, a test session was conducted. In the test session object, *Y* was replaced with object *Z*, and each mouse was allowed to explore the two objects for 10 min (Figures 3(c) and 3(d)). The interaction session was recorded, and time was noted when the animal touched its nose to any of the two objects for at least 1 sec. Discrimination index (DI) was determined by using the following formula:

(2) $$\begin{array}{c} \text{DI}=\frac{\text{Time spent with the novel object}}{\text{Total time spent with both objects}}\times 100. \end{array}$$

### 2.6. Elevated Plus Maze Test

An elevated plus (+) shaped platform with two open arms and two closed arms was used for this test. Mice were placed in the junction of the arms facing one of the closed arms. Mice movement was recorded for 10 min (Figures 3(e) and 3(f)). A mouse was considered in an arm when its head, forelimbs, and more than half of its body were in the respective arm. The amount of time spent in the open and closed arms was noted for each mouse [54].

### 2.7. Tail Suspension Test

Mice were suspended inverted by their tails at an elevated platform for 6 min (Figure 3(g)). The struggle and resting behaviors of each of the mice were recorded and observed. For each mice time, it spent struggling was noted and analyzed [55].

### 2.8. Statistical Analysis

GraphPad Prism (Version 5.01) software was used to analyze results via *T* test and One Way ANOVA. For significance, the *P* value cutoff was set at 0.05. Post hoc analysis was done using Bonferroni's post hoc test. Data are presented here as mean ± standard error mean.

## 3. Results

### 3.1. Computational Analyses

**Figure 4:**
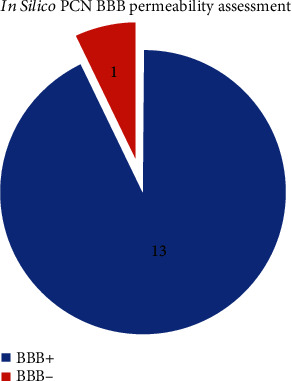
Pyocyanin BBB permeability simulations. Most simulations predicted PCN to be BBB permeable/active.

PCN is a small tricyclic compound with a monoisotopic mass of 210.079 g/mol. The chemical and structural information obtained from Pubchem and ChEMLB is given in Table 1. Four prediction servers were employed for estimating the BBB permeability of PCN. The blood-brain barrier prediction server calculated 8 predictions for PCN using SVM and AdaBoost algorithms and four molecular fingerprints (MACCSFP, OpenbabelFP2, Molprint2DFP, and PubChemFP). Each algorithm used each molecular fingerprints and the thresholds were set at default. The prediction using the AdaBoost algorithm with Molprint2DFP generated a PCN score lower than the threshold, while the other seven predicted PCN to be BBB permeable Table 2. PreADMET server computed in vivo blood-brain barrier penetration (*C.brain/C.blood*) score of 0.735186 while Way2Drug server provided LogBB value for PCN to be 0.333 based on structure and smiles, respectively. According to BB (*C.brain/C.blood*) score PCN had middle absorption while based on LogBB value PCN was highly absorbable into CNS. PreADMET also predicted PCN to have 95.08% human intestinal absorption, in vitro, Madin Darby Canine Kidney cell permeability was 216.14 nm/sec and in vitro Caco2 (human colorectal carcinoma) cell permeability 24.466 nm/sec (Middle permeability threshold 4~70). Plasma protein bonding potential was predicted to be 76.22% (the threshold for weakly bounded chemicals <90%). SwissADME server qualitatively characterized PCN as BBB permeable compound based on the BOILED-egg model. SwissADME also classified PCN to have high gastrointestinal absorbance. PCN was predicted to be an inhibitor for CYP1A2 and CYP3A4 by SwissADME. All in silico BBB permeability simulation results are summarized in Figure 4.

### 3.2. In Vivo Verification of PCN BBB Permeability

**Figure 5:**
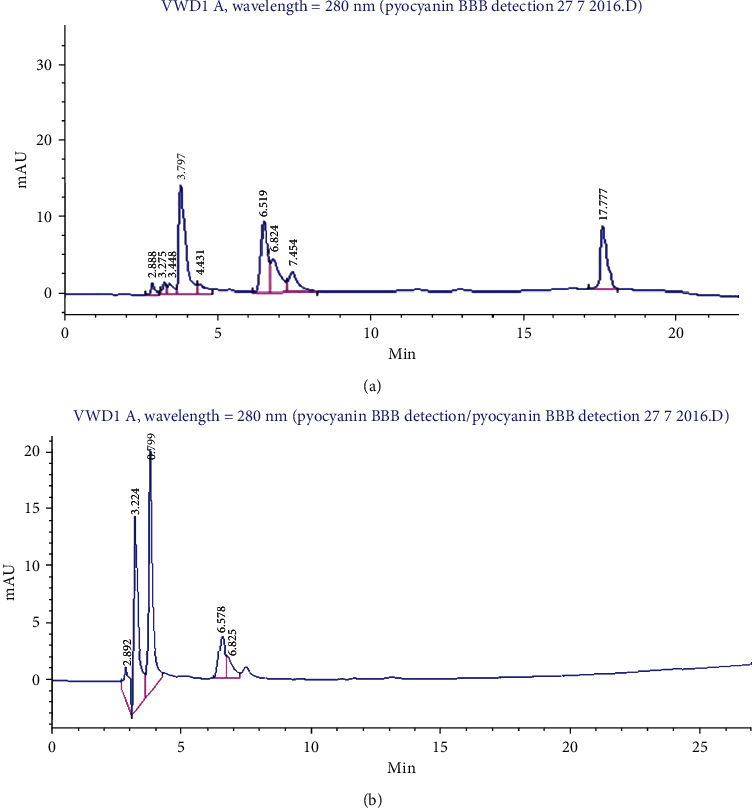
HPLC confirmation of pyocyanin BBB permeability. (a) HPLC analysis of murine brain homogenate with intraperitonial PCN injection and (b) control murine brain homogenate without PCN injection.

PCN BBB permeability and induction of oxidative stress assessment were conducted using a bi-prong validation strategy. HPLC analysis was performed on homogenized harvested brain samples of control and test groups, 12 hours post IP injection (Figure 5). PCN was detected at 17.461 RT, thus indicating permeability across the BBB.

**Figure 6:**
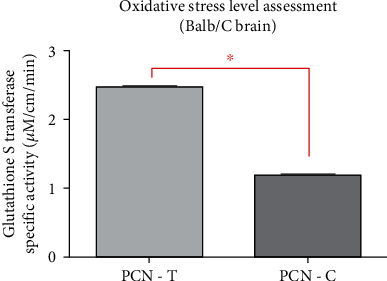
PCN redox activity assessment across BBB. Experimental mice with PCN IP injection exhibited about~2.54 *μ*M/cm/min enzyme activity in comparison to ~1.20 *μ*M/cm/min enzyme activity for control mice.

Glutathione S-transferase enzyme activity was observed as a measure of oxidative levels in test and control animals. Redox bioactivity was assayed in mice (*n* = 3) preinjected with PCN (24 hrs) and euthanized. The brains were obtained and homogenized. Total proteins were estimated and a standard curve was developed. Enzymatic activity of glutathione S-transferase (GST) was calculated to the total protein content of the test (PCN injected) and control animals (Supplementary Table 1). Results suggested a nearly two-fold increase in GSH-CDNB conjugate levels in the brains of PCN injected mice in comparison to control (Figure 6). Thus exhibiting redox activity across the BBB (*P* value < 0.0001).

### 3.3. PCN-Induced Neurotoxicity

Prolong PCN exposure has been more detrimental than short-termed high doses in all of the cognitive functions and phenomena studied in this study including memory formation, memory retrieval, object recognition, social preference, and induction of anxiety and depression.

#### 3.3.1. PCN Impact on Spatial Memory Learning

**Figure 7:**
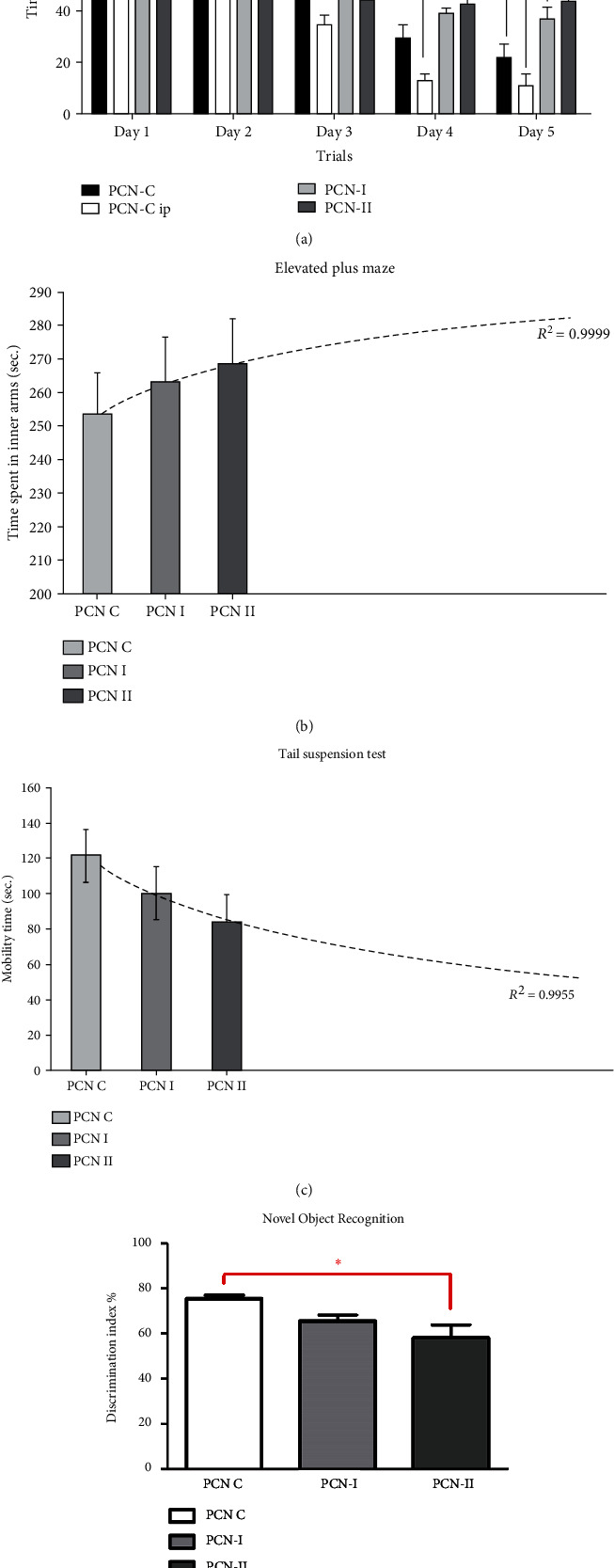
(a) Murine behavioral tests for assessment of potential PCN-induced impairment of cognitive functions. Morris water maze test was used to assess memory development in Balb/C mice. PCN-C and PCN-C ip (*n* = 8) were control with single and 5 day saline injections, respectively, PCN-I and II were test groups (*n* = 8 each). Mice were trained for 5 days and tested on the 6th. The time required to reach the destination was recorded and analyzed. At day 4, the mice took (PCN-C) 29.65 sec, (PCN-C ip) 12.9 sec, (PCN-I) 39.1 sec, and (PCN-II) 42.9 sec. While on the fifth day, the time recorded was 21.92, 10.9, 36.9, and 43.97 seconds, respectively. At day 4, PCN-I and PCN-II took significantly more time for reaching the destination in comparison to PCN-C ip (^##^*P* value < 0.005), while on day 5, both the test groups took greater time to complete the task. Hence, indicating hampered memory formation in comparison to control group. Data is presented as mean ± SEM and analyzed with two way ANOVA followed by Bonferroni's post hoc test (^∗^*P* value < 0.005 compared to PCN-C, ##*P* value < 0.005, and ###*P* value < 0.001 compared to PCN-C ip). (b) Murine behavioral tests for assessment of potential PCN-induced impairment of cognitive functions. Balb/C mice were subjected to the elevated plus maze with two open arms and two closed ones for assessing anxiety patterns. The time spent in each group of arms was recorded and analyzed. Group PCN-II spent slightly more time in open arms in comparison to PCN-C and PCN-I (*P* value > 0.5). The impact of extended PCN exposure was estimated using *y* = 13.982ln(x) + 253.37 (the calculated *R*^2^ = 0.9999 is representing good fit of data in the prediction of extended exposure of PCN). (c) Murine behavioral tests for assessment of potential PCN-induced impairment of cognitive functions. Tail suspension test studies depression patterns in stranded situations. Mice were suspended inverted via their tails. The time spent struggling was noted and analyzed. Group PCN-II spent slightly less time struggling to correct their inverted position in comparison to PCN-C and PCN-I (*P* value > 0.5). The impact of extended PCN exposure was estimated using *y* = −33.7ln(x) + 122.13 (the calculated *R*^2^ = 0.9955 is representing good fit of data in the prediction of extended exposure of PCN). (d) Murine behavioral tests for assessment of potential PCN-induced impairment of cognitive functions. Group PCN-II were relatively unable to discriminate between the two objects presented during the trials in comparison to PCN-C and PCN-I. The significant difference was observed between PCN-C and PCN-II (*P* value < 0.5).

Learning ability was assessed in mice with a focus on spatial memory formation using the Morris water maze test. The time mice took to reach the hidden platform shortens with each training day as shown in Figure 7(a). PCN-I group mice since day 3 of training, a clear pattern emerged between the four groups; control mice were taking less time to reach the platform in comparison to PCN-I and PCN-II mice, on day 4 and day 5, PCN-I (37 ± 10.3 sec) (*P* < 0.005) and PCN-II (44 ± 5.62 sec) (*P* < 0.001), the time difference was significant in contrast to PCN-C (22 ± 11.3 sec) and PCN-C ip (10.9 sec). Longer exposure of PCN significantly impaired the spatial memory of platform location in mice (Supplementary Table 2).

#### 3.3.2. PCN Impact on Anxiety and Depression

To identify the effect of PCN on anxiety, the elevated plus maze was performed. PCN increased anxiety in mice in a dose-dependent manner by increasing the time spent in the closed arm of the maze. Group PCN-II spent slightly more time in open arms in comparison to PCN-C and PCN-I (*P* value > 0.5). The impact of extended PCN exposure was estimated using:

(3) $$\begin{array}{c} y=13.982\ln\left(\text{x}\right)+253.37. \end{array}$$

The calculated *R*^2^ = 0.9999 is representing the good fit of data in the prediction of extended exposure of PCN (Figure 7(b)).

Tail suspension test is used to identify depression in rodents. Pyocyanin induced depression in a dose-dependent manner evident by a reduction in struggling time (Supplementary Table 4). PCN-I displayed lesser struggling time than control during the tail suspension, and PCN-II further reduced this time by increasing depression (*P* value > 0.5). The impact of extended PCN exposure was estimated using:

(4) $$\begin{array}{c} y=-33.7\ln\left(\text{x}\right)+122.13. \end{array}$$

The calculated *R*^2^ = 0.9955 is representing the good fit of data in the prediction of extended exposure of PCN (Figure 7(c)).

#### 3.3.3. Pyocyanin Impact on Object Recognition

Novel object recognition test was performed to assess the influence of pyocyanin on the hippocampus and rhinal cortices. All the groups showed discrimination towards a novel object by spending more than 50% of total interaction time with the novel object compared to the control (Supplementary Table 5). However, the discrimination index of PCN-II was significantly lower than the control (*P* < 0.05) (Figure 7(d)).

## 4. Discussion

In this study, we attempted to investigate PCN permeability across the BBB using a comprehensive set of computational and in vivo analytical techniques and examined potential neurotoxic consequences in animal models. BBB is a highly regulated structure that separates blood and CNS fluids and controls all of the movement into and out of the brain [56, 57]. BBB serves as a protective shield against a wide majority of invading pathogens [58]. Yet, molecules with appropriate physicochemical properties are capable of crossing this barricade [59, 60]. Although *P. aeruginosa* is a rare cause of meningitis and infection in CNS, however hospitalized patients receiving intensive care have been reported to acquire nosocomial infections occasionally coupled with complications as well [61–63]. PCN is the main virulence deterrent possessed by *P. aeruginosa* with the potential to inflict multiple different damages to the host due to its redox-active nature [64]. Cytotoxic effects have been studied for PCN in CNS cell lines which indicate its potential neurotoxicity [65, 66]. While some studies in invertebrates have shown the probability of neurodegenerative effects [67]. PCN's ability to target mitochondrial redox pathways for inflicting cytotoxicity [68] raises the concern due to the involvement of ROS originating from mitochondria in neurodegenerative disorders and aging [69–71]. Keeping in view the high diffusion potential across the biological membranes and hydrophilic and hydrophobic solubility nature of PCN, we hypothesized its permeability into the brain across BBB and consequent neurotoxicity due to induction of oxidative stress.

Computational approaches adopted for predicting permeability were based on a wide range of algorithms. Using diverse chemical and structural information for computing PCN movement provided a sound basis for proceeding to animal model studies. Out of 14 in silico approaches, 13 predicted PCN to be permeable across BBB. This permeability could be ascribed to its physiochemical and structural properties [60]. PCN is a small tricyclic compound structurally very similar to methyl blue (which is a thio-analog) [72].  Table 1 summarizes the key structural features of PCN indicating it as a smaller molecule. Interestingly, tricyclic antidepressants (TCA), a class of CNS medication, shares key structural features with PCN. TCA (i.e., clomipramine and imipramine) are able to cross BBB [37]. This structural similarity between PCN and TCA provided the ground work for this study (Figure 1(b)).

The blood-brain barrier predictor server computes permeability of any query compound using support vector machine (SVM) and AdaBoost algorithms with help of four sets of fingerprints based on reported data of 1593 compounds [73]. SVM algorithm with all the four fingerprints predicted PCN as BBB permeable. SVM algorithms have been reported to be more accurate in their computation for predicting BBB permeability [35]. PreADMET server uses [74] classification for assessing the central nervous system activity of a given structure [74]. The cutoff for BBB permeability are *BB (C.brain/C.blood)* 0.40 and LogBB -0.4, thus predicting PCN to be CNS active. The degree of absorption was also estimated using Ma X. et al. 2005 classification. The cutoff values for *BB (C.brain/C.blood)* were high absorption > 2.0, middle absorption 0.1~2.0, and low absorption to CNS was <0.1, while for log BB values were >0.3 for high, −1.0~0.3 for middle, and < −1.0 for low absorption [75]. PreADMET showed that PCN also satisfies the Lipinski rule of five (RO5). According to this rule, a compound with molecular weight < 500, Clog *P* < 5, H − bond donors < 5, and H − bond acceptors < 10 is more likely to show good absorption and permeability across biological membranes [76]. SwissADME uses the “BOILED-egg” (Brain or intestinal estimated permeation predictive model) model for predicting HIA and BBB permeability. This passive absorption model was developed [77]. This model is based on an earlier attempt by [78] who discriminated between poorly absorbed and well-absorbed molecules based on their physicochemical and structural properties, i.e., polar surface area, polarity, and lipophilicity [78].

We used a higher dosage of PCN in the murine models for the reason to amplify the potentially toxic effects for better understanding. PCN was detected in harvested brain tissues of balb-c mice 4 hrs post IP injection. A minute quantity of PCN was observed in tissue homogenate during the extraction process when homogenate was acidified it changed its color to a more reddish shade as discussed [20]. HPLC provided conclusive proof for penetration of PCN in the mice brain after IP injection. PCN extracted brain tissue homogenate was analyzed as described and exhibited similar retention patterns [51, 79]. The redox activity was studied using GSH enzyme assay [52]. Brain homogenates of mice injected with PCN exhibited a nearly two-fold increase in GSH enzyme activity in comparison to the control animals. To correlate this induction of oxidative stress, the lungs and liver homogenates were also tested for induction of oxidative stress [80]. The oxidative stress levels in the lungs and liver were per reported studies, i.e., high ROS concentrations in the lungs [13, 27] and lower in the liver [20, 81, 82].

PCN-impacted cognition as observed in animal behavior tests. PCN continuous exposure affected the spatial memory in mice of group PCN-II which received continual PCN injections for five days. Control group mice showed better spatial learning relative to both groups receiving a PCN dose. While PCN-I animals took more time in finding the submerged platform than control animals but were better than PCN-II animals which on the last day of 5-day training were having trouble finding the hidden platform. Morris water maze is a key tool to assess spatial cognitive function in CNS injuries and neurodegenerative disorders, i.e., Alzheimer's disease [83, 84]. Spatial learning and Morris water maze performance rely on a functionally integrated neural network comprising of coordinated actions of different brain regions, i.e., the hippocampus, striatum, basal forebrain, cerebellum, and cerebral cortex along with neurotransmitter systems [83]. Loss of spatial memory is reported in neurological disorders, i.e., Parkinson's disease [85].

The onset of anxiety and depression is an important neurological and psychiatric development in the course of disease progression and impacts patients' behavior towards treatment [86]. Commensal or invading microbes both have been known to influence the host's neuronal interactions and pathways [87–89]. Elevated plus maze exploits the rodents' intrinsic dislike for elevation and open areas and exploratory behavior in new and different environments. The exploratory behavior of the mice in both open and closed arms indicates the anxiety levels [90]. Similarly, the tail suspension test explores the inescapable stress due to suspended inverted by tails. The development of an immobile posture in the trapped situation indicates depression [91]. The difference in anxiety and depression levels exhibited by the three experimental groups was insignificant. Although there was an increasing trend with an increase in PCN exposure (PCN-II). When extended, this trend might result in a significant impact on the host. Although these neurological disorders could not be associated with “a single factor” or “in an isolated manner,” yet studies have reported the onset of anxiety and depression in patients suffering from chronic diseases, i.e., cystic fibrosis [92], chronic obstructive pulmonary disease [93], bacterial [94], and viral [95] infections as well. Patients with cystic fibrosis, burnt patients, and urinary tract infections have been reported to exhibit anxious and depressive behaviors [96, 97]. Such clinical situations are commonly coupled with *P. aeruginosa* infection as a primary disease complication, but there has been no such study to evaluate the probable linkage between *P. aeruginosa* infection and depression and anxiety. Interestingly, in another study unrelated to cystic fibrosis, pulmonary infection, and bronchial colonization by *P. aeruginosa* has been reported to cause such psychotic disorders with more bias towards female patients [98]. Despite the fact that the study did not observe the degree of PCN secretion as a variable during the infection and colonization, the role of PCN is well established in *P. aeruginosa* pulmonary infections [13].

Novel object recognition test is used to study human amnesia in animal models by observing the animal's ability to recognize and respond to a new stimulus [99]. This memory test relies on rodents' exploratory behavior without any external interference [100]. PCN-II animals exhibited significantly reduced preference towards the novel object in contrast to control animals. While PCN-I animals who received a single high dose also exhibited reduced preference insignificantly. The impact of exposure duration could be the key in such situations. Oxidative stress is a known factor in neurological disorders, i.e., amnesia [101]. A probable linkage between *P. aeruginosa* infection and retrograde amnesia was reported in a hospitalized patient [102]. The patient was admitted for hemolytic streptococcus infection which got complicated with respiratory arrest due to bilateral pneumonia caused by *P. aeruginosa*. The patient exhibited short-term memory loss and was unable to recall the events of his period of stay in the hospital. It is difficult to consider *P. aeruginosa* or PCN as a sole reason for this situation as the streptococci were also isolated from crebro-spinal fluid, yet it sheds some light on probable linkage between the two. These results exhibit a similar behavioral pattern as reported by the recently published study focusing on murine behaviors, i.e., locomotor activity and immobility behavior [31].

## 5. Conclusions

We have demonstrated the BBB permeability of PCN and also its ability to influence the cognitive functions of the test animals. In the light of these results, persistent and chronic nosocomial infections caused by *P. aeruginosa* pose a greater threat not only to the physiological but psychological as well as neurological wellbeing of the patients. This is the first study exploring the potential role of PCN in influencing the cognitive functions of test animals.

## Figures and Tables

**Table 1:** Structural information of pyocyanin.

Name	Pyocaynin
Molecular weight	210.23
Number of hydrogen bond acceptors	2
Number of hydrogen bond donors	0
Number of atoms	16
Number of bonds	18
Number of rings	3
Number of rotable bonds	0
Total charge	0
Molecular polar surface area	34.89

**Table 2:** The blood-brain barrier prediction server predictions for PCN BBB permeability. Total 8 simulations were run for evaluating the BBB permeability potential prediction for PCN. Only simulation no. 3 (using AdaBoost algorithm and Molprint2DFP molecular fingerprints) predicted PCN to be unable to cross BBB due to its generated score (−2.882) lesser than the default threshold.

Sr. no.	Algorithm	Molecular finger prints	PCN BBB score	Threshold of BBB−/BBB+	Result
1	AdaBoost	MACCSFP	5.435	0	BBB+
2	AdaBoost	OpenbabelFP2	11.656	0	BBB+
3	AdaBoost	Molprint2DFP	−2.882	0	BBB-
4	AdaBoost	PubChemFP	6.876	0	BBB+
5	SVM	MACCSFP	0.069	0.02	BBB+
6	SVM	OpenbabelFP2	0.159	0	BBB+
7	SVM	Molprint2DFP	0.33	0	BBB+
8	SVM	PubChemFP	0.244	0	BBB+

## Data Availability

Authors have provided their data in the Supplementary Information files alongside their manuscript.

## Supplementary Materials

Supplementary 1 Supplementary Table 1: raw data of glutathione S-transferase activity assay.

Supplementary 2 Supplementary Table 2: raw data of Morris water maze assay for assessment of memory in test animals.

Supplementary 3 Supplementary Table 3: raw data of elevated plus maze activity assay for assessment of anxiety in test animals.

Supplementary 4 Supplementary Table 4: raw data of tail suspension test for assessment of depression in test animals.

Supplementary 5 Supplementary Table 5: raw data of novel object recognition assay for assessment of memory in test animals.
