# Supplementary material for: Evaluation of Blood-Brain-Barrier Permeability, Neurotoxicity, and Potential Cognitive Impairment by Pseudomonas aeruginosa's Virulence Factor Pyocyanin
Source: Oxid Med Cell Longev. 2022 Mar 17;2022:3060579. doi: 10.1155/2022/3060579 (PMC8948603; doi:10.1155/2022/3060579)

Organs	brain (PCN ip)	brain (control)
OD (340nm)	0.116	0.051
	0.11	0.053
	0.121	0.057
	0.119	0.057
	0.118	0.057
	0.119	0.058
	0.123	0.057
	0.121	0.058
	0.12	0.059
	0.119	0.057
mM/cm	0.012083333	0.0053125
	0.011458333	0.005520833
	0.012604167	0.0059375
	0.012395833	0.0059375
	0.012291667	0.0059375
	0.012395833	0.006041667
	0.0128125	0.0059375
	0.012604167	0.006041667
	0.0125	0.006145833
	0.012395833	0.0059375
Specific Activity (mM/cm/min)	2.417	1.063
	2.292	1.104
	2.521	1.188
	2.479	1.188
	2.458	1.188
	2.479	1.208
	2.563	1.188
	2.521	1.208
	2.5	1.229
	2.479	1.188

Protein BSA standard curve						
BSA ug/10	OD 280 nm					Average
5	0.035	0.037	0.037	0.039	0.039	0.0374
10	0.063	0.064	0.065	0.066	0.068	0.0652
15	0.104	0.106	0.106	0.108	0.109	0.1066
20	0.137	0.138	0.138	0.14	0.14	0.1386
25	0.173	0.173	0.174	0.175	0.176	0.1742
30	0.207	0.209	0.21	0.212	0.215	0.2106
35	0.25	0.251	0.253	0.254	0.256	0.2528
40	0.279	0.279	0.281	0.282	0.282	0.2806
45	0.293	0.294	0.298	0.299	0.299	0.2966
50	0.364	0.365	0.366	0.368	0.37	0.3666

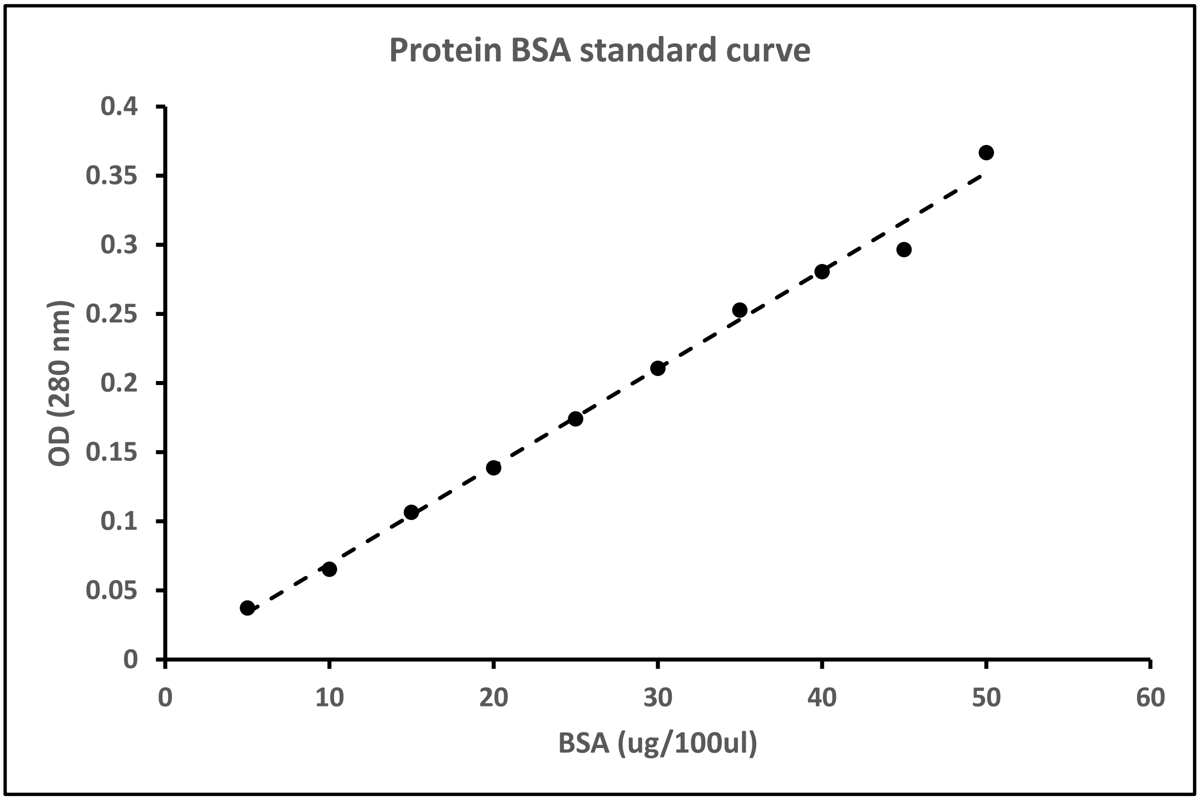

Supplement: Supplementary 1 — Supplementary Table 1: raw data of glutathione S-transferase activity assay. [file 3060579.f1.pdf]
