# Supplementary material for: Evaluation of Blood-Brain-Barrier Permeability, Neurotoxicity, and Potential Cognitive Impairment by Pseudomonas aeruginosa's Virulence Factor Pyocyanin
Source: Oxid Med Cell Longev. 2022 Mar 17;2022:3060579. doi: 10.1155/2022/3060579 (PMC8948603; doi:10.1155/2022/3060579)
Supplement: Supplementary 2 — Supplementary Table 2: raw data of Morris water maze assay for assessment of memory in test animals. [file 3060579.f2.pdf]

Day 1		PCN-C				
test subjects	T1	T2	T3	T4	T5	
C1	90	90	90	90	90	
C2	4	46	7	69	72	
C3	90	90	38	14	90	
C4	12	63	90	72	85	
C5	90	90	61	67	86	
C6	90	90	90	90	90	
Average	62.66667	78.16667	62.66667	67	85.5	71.2
Standard Deveation						10.20172
Day 2		PCN-C				
test subjects	T1	T2	T3	T4	T5	
C1	90	90	90	90	90	
C2	90	90	6	22	31	
C3	49	90	39	45	16	
C4	90	45	44	48	38	
C5	82	72	44	48	38	
C6	90	47	42	35	15	
Average	81.83333	72.33333	44.16667	48	38	56.86667
Standard Deveation						19.09457
Day 3		PCN-C				
test subjects	T1	T2	T3	T4	T5	
C1	52	20	90	90	90	
C2	17	4	4	77	10	
C3	57	24	90	60	90	
C4	90	90	49	76	70	
C5	61	29	50	76	70	
C6	90	6	15	80	90	
Average	61.16667	28.83333	49.66667	76.5	70	57.23333
Standard Deveation						18.79812
Day 4		PCN-C				
test subjects	T1	T2	T3	T4	T5	
C1	39	49	6	29	90	
C2	3	3	2.6	3	7	
C3	50	5	5	10	7	
C4	90	90	12	33	30	
C5	41	32	12	32	31	
C6	24	10	34	90	19	
Average	41.16667	31.5	11.93333	32.83333	30.66667	29.62
Standard Deveation						10.73661
Day 5		PCN-C				
test subjects	T1	T2	T3	T4	T5	
C1	24	24	3	14	11	
C2	10	3	26	4	11	
C3	32	5	4	24	19	
C4	90	55	15	12	15	
C5	40	25	15	13	16	

C6	45	40	28	8	24	
Average	40.16667	25.33333	15.16667	12.5	16	21.83333
Standard Deveation						11.33517

Day 1		PCN-I				
test subjec	T1	T2	T3	T4	T5	
G1	90	90	47	90	90	
G2	90	90	90	90	54	
G3	90	90	90	90	90	
G4	90	90	90	90	90	
G5	90	90	3	90	90	
G6	90	90	25	90	89	
Average	90	90	57.5	90	83.83333	82.26667
						14.10014

Day 2		PCN-I				
test subjec	T1	T2	T3	T4	T5	
G1	90	10	90	90	90	
G2	90	90	68	14	37	
G3	15	45	12	15	33	
G4	90	62	90	90	90	
G5	90	90	17	9	90	
G6	90	20	55	15	43	
Average	77.5	52.83333333	55.33333333	38.83333	63.83333	57.66667
						14.27313

Day 3		PCN-I				
test subjec	T1	T2	T3	T4	T5	
G1	25	21	72	18	10	
G2	90	14	61	30	31	
G3	18	15	31	7	6	
G4	64	90	90	90	90	
G5	90	90	90	90	90	
G6	10	26	21	17	73	
Average	49.5	42.66666667	60.83333333	42	50	49
						7.589283

Day 4		PCN-I				
test subjec	T1	T2	T3	T4	T5	
G1	11	5	12	11	10	
G2	22	44	60	9	44	
G3	6	4	18	5	11	
G4	50	90	90	90	50	
G5	90	90	90	90	90	
G6	20	13	17	6	25	
Average	33.16666667	41	47.83333333	35.16667	38.33333	39.1
						5.725916

Day 5		PCN-I				
test subjec	T1	T2	T3	T4	T5	
G1	8	5	3	4	3	
G2	16	20	40	24	15	
G3	14	8	7	15	11	
G4	22	90	90	90	15	
G5	90	90	90	90	90	

G6	25	90	13	17	12	
Average	29.16666667	50.5	40.5	40	24.33333	36.9
						10.31086

Day 1		PCN-II				
test subjec	T1	T2	T3	T4	T5	
G1	25	90	90	90	16	
G2	90	90	90	90	90	
G3	35	90	37	79	90	
G4	90	90	90	90	90	
G5	90	90	10	17	90	
G6	90	90	90	90	33	
Average	70	90	67.83333	76	68.16667	74.4
						9.317397

Day 2		PCN-II				
test subjec	T1	T2	T3	T4	T5	
G1	90	4	12	42	45	
G2	90	90	90	90	90	
G3	50	8	90	90	90	
G4	90	90	90	90	90	
G5	25	8	45	5	22	
G6	90	90	90	38	90	
Average	72.5	48.33333	69.5	59.16667	71.16667	64.13333
						10.27835

Day 3		PCN-II				
test subjec	T1	T2	T3	T4	T5	
G1	13	7	17	18	30	
G2	90	90	86	90	90	
G3	7	52	5	36	27	
G4	3	90	90	90	90	
G5	26	7	15	14	5	
G6	90	35	76	32	15	
Average	38.16667	46.83333	48.16667	46.66667	42.83333	44.53333
						4.076355

Day 4		PCN-II				
test subjec	T1	T2	T3	T4	T5	
G1	18	6	7	13	7	
G2	90	6	68	58	28	
G3	45	60	26	22	90	
G4	90	90	90	90	90	
G5	9	4	8	3	2	
G6	90	40	89	34	14	
Average	57	34.33333	48	36.66667	38.5	42.9
						9.439574

Day 5		PCN-II				
test subjec	T1	T2	T3	T4	T5	
G1	5	6	3	45	5	
G2	55	90	83	90	90	
G3	17	20	15	15	32	
G4	90	90	90	90	90	
G5	6	26	8	7	40	

G6	40	56	54	34	27	
Average	35.5	48	42.16667	46.83333	47.33333	43.96667
						5.261759
