# Supplementary material for: Evaluation of Blood-Brain-Barrier Permeability, Neurotoxicity, and Potential Cognitive Impairment by Pseudomonas aeruginosa's Virulence Factor Pyocyanin
Source: Oxid Med Cell Longev. 2022 Mar 17;2022:3060579. doi: 10.1155/2022/3060579 (PMC8948603; doi:10.1155/2022/3060579)
Supplement: Supplementary 3 — Supplementary Table 3: raw data of elevated plus maze activity assay for assessment of anxiety in test animals. [file 3060579.f3.pdf]

Groups	Subjects	inside time (sec)
PCN C	C1	228
	C2	270
	C3	265
	C4	267
	C5	240
	C6	250
	C7	259
	C8	246
PCN I	T1	265
	T2	294
	T3	250
	T4	241
	T5	253
	T6	276
	T7	280
	T8	240
PCN II	T1	280
	T2	262
	T3	274
	T4	254
	T5	244
	T6	298
	T7	261
	T8	257
