# Supplementary material for: Evaluation of Blood-Brain-Barrier Permeability, Neurotoxicity, and Potential Cognitive Impairment by Pseudomonas aeruginosa's Virulence Factor Pyocyanin
Source: Oxid Med Cell Longev. 2022 Mar 17;2022:3060579. doi: 10.1155/2022/3060579 (PMC8948603; doi:10.1155/2022/3060579)
Supplement: Supplementary 4 — Supplementary Table 4: raw data of tail suspension test for assessment of depression in test animals. [file 3060579.f4.pdf]

Groups	Subjects	exertion (Sec)
	C1	139
	C2	116
	C3	107
PCN C	C4	109
	C5	134
	C6	91
	C7	115
	C8	125
	T1	149
	T2	67
	T3	70
PCN I	T4	124
	T5	61
	T6	100
	T7	130
	T8	99
	T1	104
	T2	88
	T3	83
PCN II	T4	57
	T5	120
	T6	90
	T7	95
	T8	100
