# Supplementary material for: Evaluation of Blood-Brain-Barrier Permeability, Neurotoxicity, and Potential Cognitive Impairment by Pseudomonas aeruginosa's Virulence Factor Pyocyanin
Source: Oxid Med Cell Longev. 2022 Mar 17;2022:3060579. doi: 10.1155/2022/3060579 (PMC8948603; doi:10.1155/2022/3060579)
Supplement: Supplementary 5 — Supplementary Table 5: raw data of novel object recognition assay for assessment of memory in test animals. [file 3060579.f5.pdf]

Groups	Subjects	% discrimination index
PCN C	C1	80
	C2	72
	C3	75
	C4	75
	C5	82
	C6	77
	C7	72
	C8	70
PCN I	T1	75
	T2	63.6
	T3	70
	T4	55
	T5	65
	T6	65
	T7	68
	T8	64
PCN II	T1	72
	T2	37
	T3	66
	T4	57
	T5	58
	T6	60
	T7	56
	T8	59
